# Supplementary material for: A Unique In Vitro Assay to Investigate ABCB4 Transport Function
Source: Int J Mol Sci. 2023 Feb 24;24(5):4459. doi: 10.3390/ijms24054459 (PMC10003010; doi:10.3390/ijms24054459)
Supplement: Supplementary file 1 [file ijms-24-04459-s001.zip › ijms-2190300-supplementary.pdf]

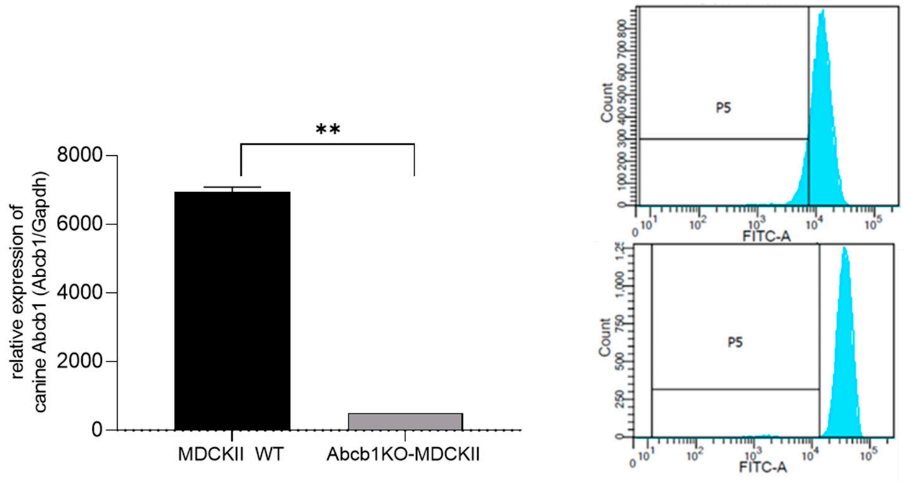

(a) **(b)**

Figure S1. Characterization of the Abcb1 knock-out MDCKII cell line.
(a) mRNA expression levels of canine Abcb1 in MDCKII parental (MDCKII WT) and Abcb1KO-MDCKII cells. Each sample's Abcb1 expression was subtracted from its Gapdh expression to determine its ΔC_T. mRNA expression levels of Abcb1 as compared to those of Gapdh as an endogenous control were determined by the formula: relative expression = $2^{(-\Delta CT)} \times 10^6$. $^{**}P < 0.01$, by Welch's t-test.
(b) Flow cytometry results (FACS) for MDCKII WT (upper panel) and Abcb1KO-MDCKII (lower panel) cells. 1×10^6 cells/mL cells were incubated with 0.1 μM calcein AM at 37 °C for 30 min. The histograms represent the geometric mean of calcein fluorescence intensity.

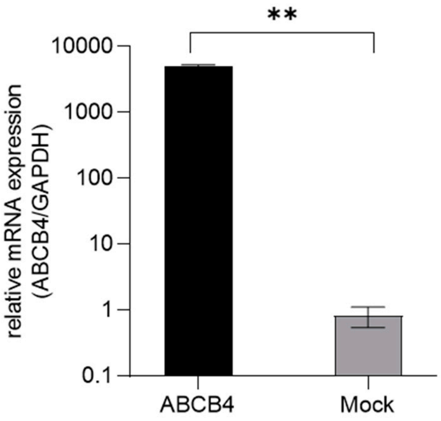

Figure S2. mRNA expression levels of ABCB4 in Abcb1KO-MDCKII-ABCB4 and Abcb1KO-MDCKII-Mock cells.

In each sample ΔC_T was calculated. mRNA expression levels of ABCB4 as compared to those of Gapdh as an endogenous control were determined by the formula: $2^{(-\Delta CT)} \times 10^6$. ** $P < 0.01$, by Welch’s t-test.

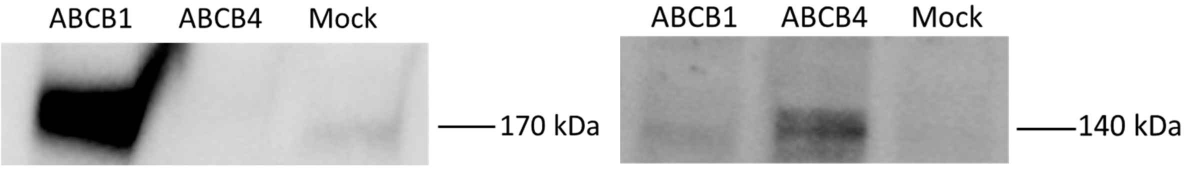

(a) **(b)**

Figure S3. Expression of human ABCB1 and ABCB4 proteins.
(a) Representative western blot results showing that a band of about 170 kDa is present in the Abcb1KO-MDCKII-ABCB1 (ABCB1) cells and absent from Abcb1KO-MDCKII-ABCB4 (ABCB4) and Abcb1KO-MDCKII-Mock (Mock) cells. Proteins were probed with primary antibody (C219, 1:1000) specific to ABCB1.
(b) Representative western blot results, showing that a band of about 140 kDa is present in the ABCB4 cells and absent from ABCB1 and Mock cells. Proteins were probed with primary antibody (P3II-26, 1:1000) specific to ABCB4.

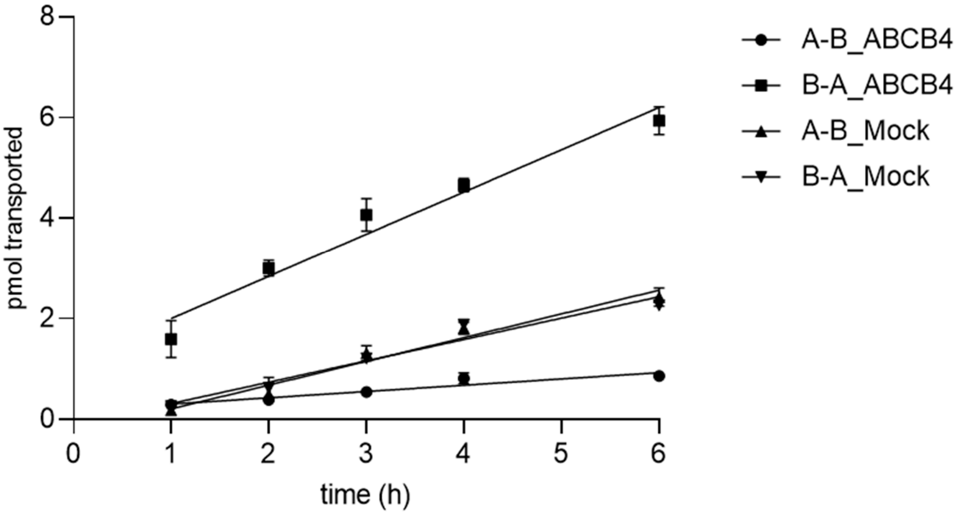

(a)

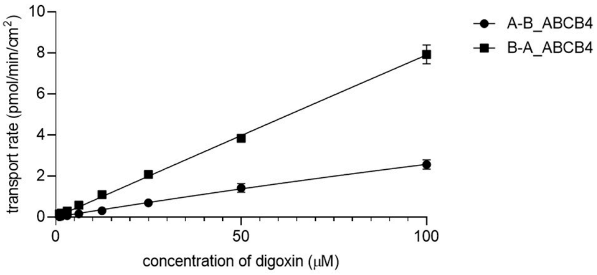

(b)

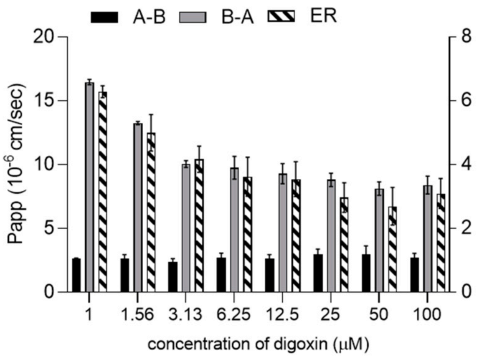

(c)

Figure S4. Transcellular transport of digoxin in the Abcb1KO-MDCKII-ABCB4 (ABCB4) and Abcb1KO-MDCKII-Mock (Mock) cells.

(a) Time course of digoxin transport. The cumulative amount of digoxin (0.1 µM, traced with 0.17 µCi/ml ^{3}H-digoxin) transported in the apical to basolateral direction (A-B) and in the basolateral to apical direction (B-A) over 6 hours in confluent cell monolayers of ABCB4 and Mock. The B-A transport of digoxin is higher in in ABCB4 cells compared to A-B transport, since ABCB4 expressed in the apical membrane of MDCKII cells and actively pumps digoxin into the apical chamber. In contrast, digoxin transport is identical in both directions in Mock cells. An incubation time of 3 hours was chosen for inhibition experiments. Data are presented as mean ± standard deviation for one representative experiment performed in triplicate. Lines are representing the linear least squares regression fit to data points (A-B_ABCB4 $p<0.05$; B-A_ABCB4, A-B_B-A_Mock $p<0.005$)

(b) Bidirectional transport of digoxin at various donor concentrations. ABCB4 cells were incubated with 1-100 µM digoxin for 180 minutes. Data are represented as means ± S.D. for three independent experiments.

(c) Papp and ER values of digoxin at various donor concentrations. ABCB4 cells were incubated with 1-100 µM digoxin for 180 minutes. Data are represented as means ± S.D. for three experiments.

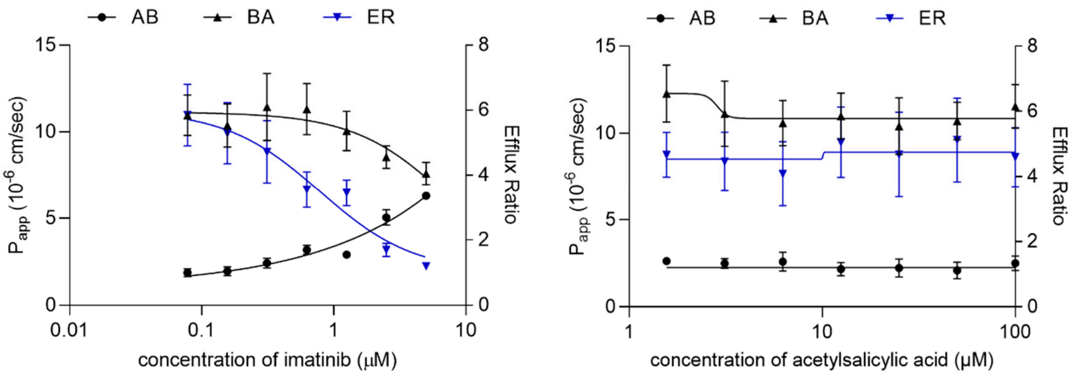

(**a**) (**b**)

Figure S5. Inhibition of digoxin transport through ABCB4 cells. (a) Inhibition of digoxin transport (Papp and ER) by imatinib; **(b)** Inhibition of digoxin transport (Papp, ER) by acetylsalicylic acid. Data are presented as mean ± standard deviation from 3 experiments each performed in triplicate.
